# Piezoelectric biomaterials for neural tissue engineering

**DOI:** 10.1002/SMMD.20230002

**Published:** 2023-04-26

**Authors:** Dongyu Xu, Hui Zhang, Yu Wang, Yuan Zhang, Fanglei Ye, Ling Lu, Renjie Chai

**Affiliations:** ^1^ State Key Laboratory of Bioelectronics Department of Otolaryngology Head and Neck Surgery Zhongda Hospital School of Life Sciences and Technology Advanced Institute for Life and Health Jiangsu Province High‐Tech Key Laboratory for Bio‐Medical Research Southeast University Nanjing China; ^2^ Department of Otology The First Affiliated Hospital of Zhengzhou University Zhengzhou Henan China; ^3^ Department of Otolaryngology Head and Neck Surgery Jiangsu Provincial Key Medical Discipline Nanjing Drum Tower Hospital The Affiliated Hospital of Nanjing University Medical School Nanjing China; ^4^ Co‐Innovation Center of Neuroregeneration Nantong University Nantong China; ^5^ Department of Otolaryngology Head and Neck Surgery Sichuan Provincial People's Hospital University of Electronic Science and Technology of China Chengdu China; ^6^ Institute for Stem Cell and Regeneration Chinese Academy of Sciences Beijing China; ^7^ Beijing Key Laboratory of Neural Regeneration and Repair Capital Medical University Beijing China

**Keywords:** biomaterials, external energy, nerve regeneration, piezoelectricity, wireless electrical stimulation

## Abstract

Nerve injury caused by trauma or iatrogenic trauma can lead to loss of sensory and motor function, resulting in paralysis of patients. Inspired by endogenous bioelectricity and extracellular matrix, various external physical and chemical stimuli have been introduced to treat nerve injury. Benefiting from the self‐power feature and great biocompatibility, piezoelectric biomaterials have attracted widespread attention in biomedical applications, especially in neural tissue engineering. Here, we provide an overview of the development of piezoelectric biomaterials for neural tissue engineering. First, several types of piezoelectric biomaterials are introduced, including inorganic piezoelectric nanomaterials, organic piezoelectric polymers, and their derivates. Then, we focus on the in vitro and in vivo external energy‐driven piezoelectric effects involving ultrasound, mechanical movement, and other external field‐driven piezoelectric effects. Neuroengineering applications of the piezoelectric biomaterials as in vivo grafts for the treatment of central nerve injury and peripheral nerve injury are also discussed and highlighted. Finally, the current challenges and future development of piezoelectric biomaterials for promoting nerve regeneration and treating neurological diseases are presented.

## Graphical abstract

Benefiting from the direct piezoelectric effect, piezoelectric biomaterials have provided various feasible strategies for wireless electric stimulation in neural tissue engineering. In this review, we will overview the recent progress in piezoelectric biomaterials for nerve neural tissue engineering, ranging from the classification and external energy‐driven piezoelectric effects to the neuro‐engineering applications of these piezoelectric biomaterials.

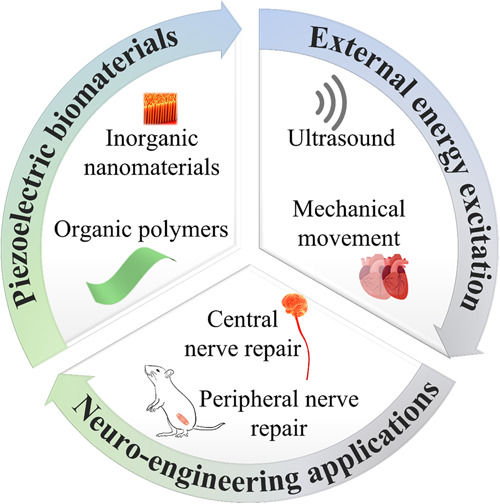

1

**Key points**

- The classification and remarkable piezoelectricity of piezoelectric biomaterials are introduced.
- The external energy‐driven piezoelectric effects of piezoelectric biomaterials in vivo are presented.
- The neuroengineering applications of piezoelectric biomaterial‐based wireless electrical stimulation devices are emphasized.

## 2 INTRODUCTION

Due to the extensive distribution of the nervous system in the human body, the nerve defects resulted from trauma or iatrogenic resection can cause the loss of sensory, motor, and other functions of patients.^1^ In order to repair damaged nerves, various clinical treatment methods have been adopted, such as end‐to‐end sutures of injured sites, autologous nerve transplantation, and nerve guidance conduits (NGCs).^2^, ^3^, ^4^, ^5^ However, autologous nerve transplantation for lager nerve defect faces challenges including donor nerve shortage and the mismatch between donor and receptor. In contrast, NGCs provide an alternative strategy and a multifunctional platform for clinical treatment of nerve injury.^6^, ^7^ In general, the endogenous electric field in the organism is an indispensable part of the biological life activities, which plays an important role in cell potential, muscle contraction, heart beating, embryonic development, and so on.^8^, ^9^ Inspired by such significant endogenous bioelectricity, external electric field stimulation (ES) has been applied to mediate neuron activity, which showed positive effects on promoting neurite elongation.^9^, ^10^, ^11^, ^12^, ^13^ However, several shortcomings have restricted application of ES such as the heavy and expensive equipment with controlled parameters for in vitro cells, the complexity of operation, and wound infection caused by in vivo electrodes. Thus, the realization of ES with portable facilities and simple operations is critical for repairing damaged tissue in clinical.

Emerging piezoelectric biomaterials with self‐powered characteristics and flexibility have been extensively developed over the past decades, providing a new idea for the application of exogenous ES in neural tissue engineering. The inherent piezoelectric effects of piezoelectric biomaterials allow them to directly convert mechanical stimuli into electrical polarization (direct piezoelectricity) without any external power supply.^14^ As a result, piezoelectric biomaterials in vivo can generate pulse voltage output under mechanical stimuli, including cell migration, mechanical movement of the body, and external energy stimulation, further stimulating surrounding cells and tissues to respond.^15^, ^16^, ^17^ Attracted by these features, much progress focused on piezoelectric biomaterial‐based wireless ES devices has been made for neural tissue engineering.

In this paper, we provide a review on piezoelectric biomaterials, which show great potential in neural tissue engineering. First, the classification of piezoelectric biomaterials involving inorganic piezoelectric nanomaterials, organic piezoelectric polymers, and their derivates is summarized, and their potential in neural tissue engineering is evaluated. Then, the external energy‐driven piezoelectric effects of piezoelectric biomaterials in vivo, including ultrasound (US)‐driven piezoelectricity, mechanical movement‐driven piezoelectricity, and other energy field‐driven piezoelectricity, are introduced in detail. After that, we focus on the applications of piezoelectric biomaterial‐based wireless ES devices in the treatment of central nerve injury (CNI), peripheral nerve injury (PNI), and other neurological diseases. Finally, we offer our opinions about the current challenges and future directions of piezoelectric biomaterials for nerve regeneration and treatment of neurological diseases.

## 3 CLASSIFICATION OF PIEZOELECTRIC BIOMATERIALS

Bioelectricity widely exists in various biological tissues, involving bones, muscles, ligaments, skin, teeth, corneas, etc., which is generally derived from low symmetrical biomacromolecules in biological tissues. These natural piezoelectric biomacromolecules can generate electrical signals under mechanical vibration to control the process of life activities, such as bone regeneration, neural repair, heart beating, and embryonic development.^18^, ^19^, ^20^, ^21^, ^22^ Thus, exogenous ES is expected to be introduced to promote damaged tissue repair. Up to date, miscellaneous flexible wireless piezoelectric biomaterials have been developed for neural tissue engineering to replace the traditional large invasive ES equipment. In this section, the classification of piezoelectric biomaterials and their derivates is provided (Table 1): (i) inorganic piezoelectric nanomaterials, and (ii) organic piezoelectric polymers.

**TABLE 1:** A summary of piezoelectric biomaterials.

Classification	Piezoelectric biomaterials	Properties	Ref
Inorganic piezoelectric nanomaterials	PZT	Adjustable mechanical strength	23, 24, 25
		High piezoelectricity	
		Leakage of Pb^2+^	
	BaTiO_3_ (BT)	Improved biocompatibility	26, 27, 28, 29
		Excellent piezoelectricity	
	BN	Anisotropy	30
		Piezoelectricity	
		Cell compatibility	
	ZnO	Piezoelectricity	31, 32, 33, 34, 35, 36, 37
		Antibacterial properties	
		Inhibitory effects at subtoxic concentrations	
Organic piezoelectric polymers	Collagen	Natural polymers	38, 39
		Piezoelectricity	
		Biocompatibility	
		Biodegradability	
	Silk	Natural polymers	40, 41, 42
		Piezoelectricity	
		Biocompatibility	
		Biodegradability	
	PVDF	Piezoelectricity	43
		Flexibility	
		Biocompatibility	
	P(VDF‐TrFE)	Higher β‐phase content	44
		Piezoelectricity	
		Flexibility	
		Biocompatibility	
	PLLA	Elasticity	45, 46, 47, 48
		Corrosion resistance	
		Low processing temperature	
		Biocompatibility	
		Biodegradability	
Piezocomposites	Inorganic piezoelectric nanomaterials/organic piezoelectric polymers	Enhanced piezoelectricity	49, 50, 51, 52
		Flexibility	
		Biocompatibility	
	Inorganic piezoelectric nanomaterials/biodegradable polymer	Piezoelectricity	53
		Flexibility	
		Biocompatibility	
		Biodegradability	
	Piezoelectric hydrogels	Piezoelectricity	54
		Soft	
		High water content	

Abbreviations: BN, boron nitride; P(VDF‐TrFE), poly(vinylidene fluoride‐trifluoro ethylene); PLLA, poly‐L‐lactic acid; PVDF, poly(vinylidene fluoride); ZnO, zinc oxide.

### 3.1 Inorganic piezoelectric nanomaterials

**FIGURE 1:**
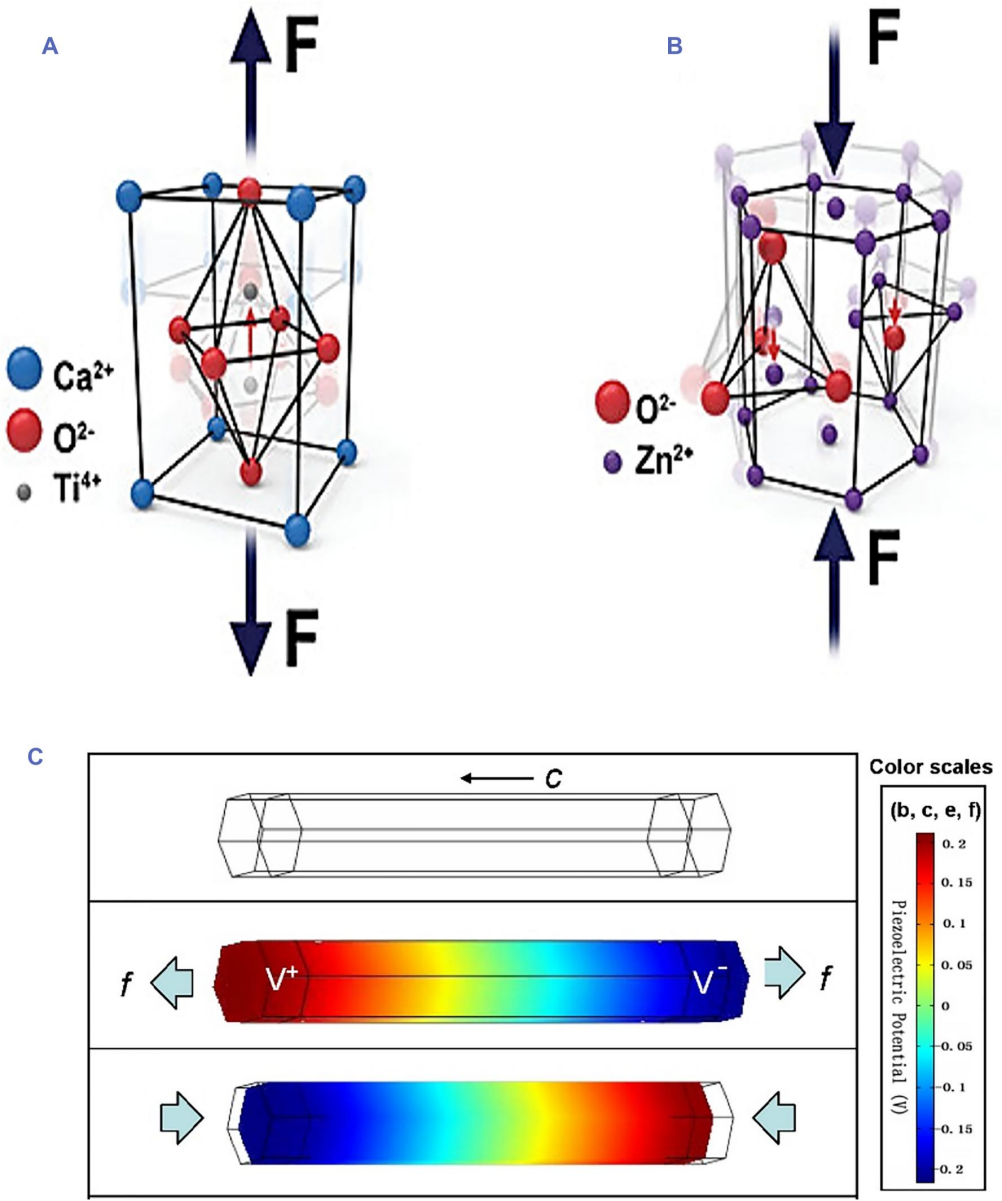
Piezoelectric characteristics of inorganic piezoelectric nanomaterials. (A) Perovskite ceramics with ABO_3_ perovskites. (B) Wurtzite crystals represented by ZnO. *Source*: (A, B) Reproduced under terms of the CC‐BY license.^23^ Copyright 2020, The Authors, published by John Wiley and Sons. (C) Piezoelectric potential distribution in a ZnO nanowire under tension and pressure. *Source*: Reproduced with permission.^61^ Copyright 2009, AIP Publishing.

Since Curie brothers discovered the piezoelectric effect of single crystal α‐quartz and Rochelle salt in 1880, efforts have been devoted on the researches of piezoelectric materials, and a variety of piezoelectric materials have been discovered and prepared.^55^, ^56^ Compared with the first discovered single crystal piezoelectric materials with narrow application areas and inefficient energy conversion, the subsequently developed single crystal or polycrystalline inorganic piezoelectric nanomaterials showed outstanding coupling coefficients, efficient signal transduction, and flexibility in multiple application scenarios. Currently, the common inorganic piezoelectric nanomaterials include perovskite ceramics with ABO_3_ perovskites,^23^, ^24^, ^57^ two‐dimensional boron nitride (BN) nanomaterials,^58^, ^59^ zinc oxide (ZnO) with P6_3_mc symmetry group crystalline structure,^59^, ^60^, ^61^, ^62^ etc. The piezoelectric properties of inorganic piezoelectric nanomaterials generate from the internal asymmetry of nanomaterial crystals. As shown in Figure 1, when subjected to mechanical stress (stretching, shearing, compression, etc.),^23^ the displacement of positive and negative ions in the crystal structure produces a dipole moment that cannot be offset by other dipoles, thus exhibiting a macroscopic potential difference.^8^

**FIGURE 2:**
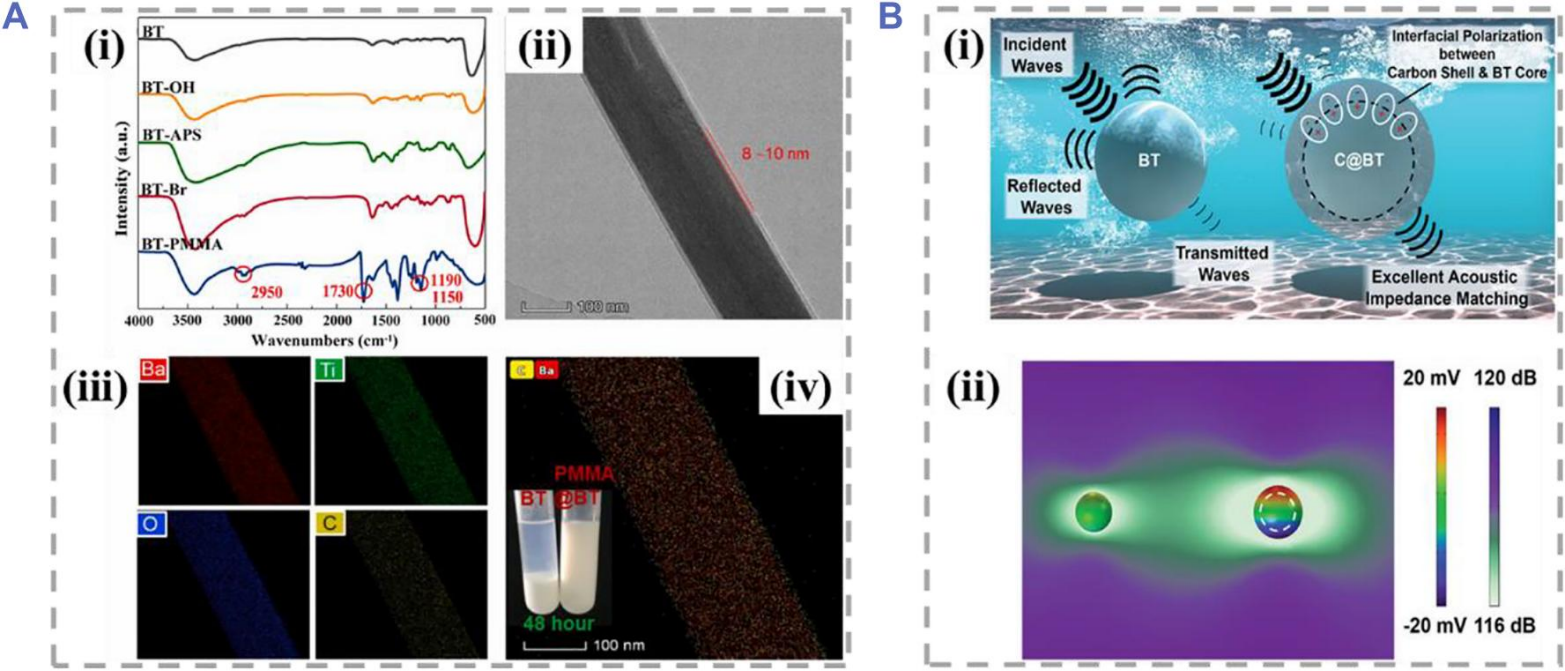
Multifunctional BT nanomaterials. (A) Fourier transform infrared spectra (i), transmission electron microscope image (ii), energy dispersive X‐ray spectroscopy mapping results (iii, iv) of PMMA@BTNWs. *Source*: Reproduced with permission.^28^ Copyright 2021, Elsevier. (B) Schematic diagram of C@BT nanoparticles used to enhance US absorption (i) and electromagnetic field distribution of the BT and C@BT nanoparticle (ii). *Source*: Reproduced with permission.^29^ Copyright 2020, John Wiley and Sons.

The lead‐based piezoelectric ceramics commercialized by Clevite Corporation as “PZT” is polycrystalline Pb(Zr, Ti)O_3_‐based solid solutions produced by doping Pb into ABO_3_ perovskites.^23^ PZT materials (PZT‐5H, PZT‐4, PZT‐8) with different mechanical strengths and high piezoelectric properties can be obtained by adjusting Zr content for various application scenarios.^23^, ^24^ However, the leakage of Pb^2+^ limits the application of PZT materials in the biomedical fields.^25^ In contrast, barium titanate (BaTiO_3_, BT) piezoelectric ceramic nanoparticles with great biocompatibility and excellent piezoelectric effects have been widely used in various fields. Some studies have shown that the ES generated by the internalized BT nanoparticles in SH‐SY5Y neuron‐like cells stimulated by external fields could promote the elongation of nerve axons and the changes of calcium and sodium fluxes, thus triggering the cell responses.^26^ In addition to promoting effect in nerve growth, BT nanoparticles possess great potential in other biomedical fields, including inducing osteoblastic differentiation of mesenchymal stem cells and tumor‐targeted therapy.^27^ Multifunctional BT nanoparticles can be constructed by various surface modification approaches of nanoparticles to improve the poor dispersion and enhance the piezoelectric performance. For example, Huang et al. produced BT nanowires coating with polymethyl methacrylate (PMMA@BTNWs), which were proved to have improved dispersion and enhanced output performance compared with BT nanoparticles, as shown in Figure 2A.^28^ Besides, a kind of BT nanoparticles with a certain thickness of carbon shell (C@BT) was proposed by Shen and coworkers, which possessed matched acoustic impedance in biological media for wireless ES therapy of Parkinson's disease (PD) (Figure 2B).^29^

Apart from the ABO_3_ perovskite piezoelectric nanomaterials, anisotropic two‐dimensional BN nanomaterials (BN nanotubes, BN nanowires, and BN nanosheets) also exhibit piezoelectric properties and excellent cell compatibility. With the US‐driven piezoelectric effect, pheochromocytoma cells (PC12 cells) treated with BN nanotubes showed increased neurites, indicating the application prospect in ES of nerve tissue engineering.^30^ Different from several inorganic piezoelectric nanomaterials mentioned above, ZnO nanomaterials exhibited inhibitory effects on nerve cells at subtoxic concentrations (10^−4^–10^−1^ μg/mL).^31^, ^32^ Therefore, the integration with polymer materials and the surface modification of nanoparticles are often used to reduce the biological toxicity of inorganic ZnO nanoparticles, and the ZnO‐based biomaterials have been used in the repair of PNI, drug delivery, and bioimaging.^33^, ^34^, ^35^ It's worth mentioning that ZnO‐based wound dressings can accelerate the healing process of chronic wounds due to the antibacterial properties and piezoelectric properties of ZnO nanomaterials.^36^, ^37^

### 3.2 Organic piezoelectric polymers

Compared with natural organic piezoelectric materials (bone, collagen, silk fibroin, and keratin),^38^, ^39^, ^40^, ^41^, ^42^ synthetic organic piezoelectric polymer materials have stronger mechanical properties and flexibility. Poly(vinylidene fluoride) (PVDF)^43^ and its derivant poly(vinylidene fluoride‐trifluoro ethylene) [P(VDF‐TrFE)] have a great attraction in nerve tissue engineering. α‐PVDF with dipole moments in the opposite direction and β‐PVDF with dipole moments in the same direction dominates in PVDF polymer chains, where the counteracted piezoelectricity and the enhanced piezoelectricity can be generated under mechanical strain, respectively.^23^, ^63^ Therefore, more β‐phase crystallization in PVDF chains is expected to enhance piezoelectricity. P(VDF‐TrFE), a copolymer of PVDF, contains higher β‐phase content, thus possessing higher piezoelectric output performance than PVDF.^49^, ^50^ Recently, Nam's group employed hydroacoustic actuation to activate the electrical signal output generated by electrospun P(VDF‐TrFE) nanofiber mats to induce multiphenotypic differentiation of neural stem cells, which could effectively regulate the function of neural stem cells and was promising for developing functional neural tissue.^44^ In addition, in order to increase the β‐phase crystallization ratio, various polarization processes are widely used to prepare PVDF and P(VDF‐TrFE) based polymer materials, involving mechanical stretching, thermal drawing, electrospinning, thermal annealing, and stepwise poling methods.^44^, ^49^, ^50^

Although PVDF and PVDF‐TrFE exhibit high piezoelectric properties and promote the growth of nerve cells, their nonbiodegradable properties limit their application in in vivo transplantation. Poly‐L‐Lactic acid (PLLA), as another synthetic organic piezoelectric polymer, possesses various merits of elasticity, corrosion resistance, low processing temperature, biodegradability, and biocompatibility.^45^, ^46^ Its piezoelectric properties are independent of the formation of specific crystal phases (αʹ, α, β and γ). Besides, the fiber diameter and surface nanotopography of PLLA nanofiber mats have different induction effects on the growth of neurons, and small fiber diameter and unsmooth fiber surface can limit the elongation and branching of neurons.^47^, ^48^ Taking advantage of the piezoelectricity and biodegradability of PLLA, Luo et al. have developed a kind of PLLA mixed NGCs for repairing PNI. The results showed that such NGCs were nontissue toxic, biodegradable, and could promote nerve regeneration and myelination.^51^

In particular, piezocomposites, usually prepared by doping inorganic piezoelectric nanomaterials into organic polymer materials, inherit the high piezoelectric output performance of inorganic ceramic nanomaterials as well as the flexibility and biocompatibility of organic polymers. Piezocomposites based on organic polymers can better integrate inorganic piezoelectric nanomaterials to prepare self‐powered NGCs, patches, microspheres, fibers, and hydrogels, which are suitable for a variety of in vivo transplantation scenarios in neural tissue engineering.^8^, ^51^ In addition, the combination of piezoelectric nanomaterials and piezoelectric polymers can also play a positive synergistic role in enhancing the piezoelectric effect. For example, Jiang and his coworkers^49^ suspended an appropriate proportion of BT nanoparticles into P(VDF‐TrFE) solution, followed by an electrospinning process. In the resultant piezocomposites, BT nanoparticles endowed them with high permittivity and were used as nucleating agents to promote β‐phase crystallization of P(VDF‐TrFE), thus further enhancing the piezoelectric performance.^51^ Furthermore, BT nanoparticles can be surface modified to strengthen the interface interaction with organic piezoelectric polymers, thereby improving the dispersion and doping density.^52^ Notably, doping piezoelectric nanomaterials into naturally degradable biomaterials can impart composite materials with great piezoelectric properties, so that degradable natural biomaterials can display self‐powered characteristics and realize implantation of ES devices in vivo without secondary damage.^53^

Ascribed to good flexibility, high water content and cross‐linked network structures, hydrogels are expected to be connected with soft neural tissues, realizing soft interaction between human and electronic devices to overcome the mechanical mismatch problem of traditional electronic devices.^54^, ^64^, ^65^, ^66^ More recently, Madden et al. discussed the piezoelectric mechanism of piezoelectric hydrogels in detail and proposed the mechanism of current and voltage generation due to the difference in the moving speed of ions (cations and anions) and solvents. The presented poly(acrylic acid‐co‐acrylamide) piezoelectric hydrogel could monitor pressure sensing and sliding tactile sensing with mechanical compliance and self‐powered characteristics, which demonstrated its advantages in soft robots and wearable devices. What is more, they applied the piezoelectric hydrogel to peripheral nerve stimulation and observed obvious electromyogram signals in the gastrocnemius muscle, suggesting self‐powered nerve regulation.^54^

## 4 EXTERNAL ENERGY‐DRIVEN PIEZOELECTRIC EFFECTS

### 4.1 Ultrasound

US is an acoustic wave with a frequency beyond human hearing range (>20 kHz),^67^ which has a wide range of applications in nature, such as echolocation of bats, ultrasonic signal communication of dolphins, etc. Because of its superiority in deep tissue penetration, US hyperthermia, mechanical vibration without radiation, and biomedical safety, US therapeutic apparatus (usually 1 and 3 MHz) has been widely used in the treatment of soft tissue injury, chronic pain, sports rehabilitation, and other biomedical fields.^68^, ^69^ In addition, US can cause internal vibration and thermal effects in tissues, so a variety of US‐driven drug release systems,^70^, ^71^ long‐term bioimaging devices,^72^ and wireless ES devices^73^, ^74^, ^75^, ^76^ have attracted extensive attention.

**FIGURE 3:**
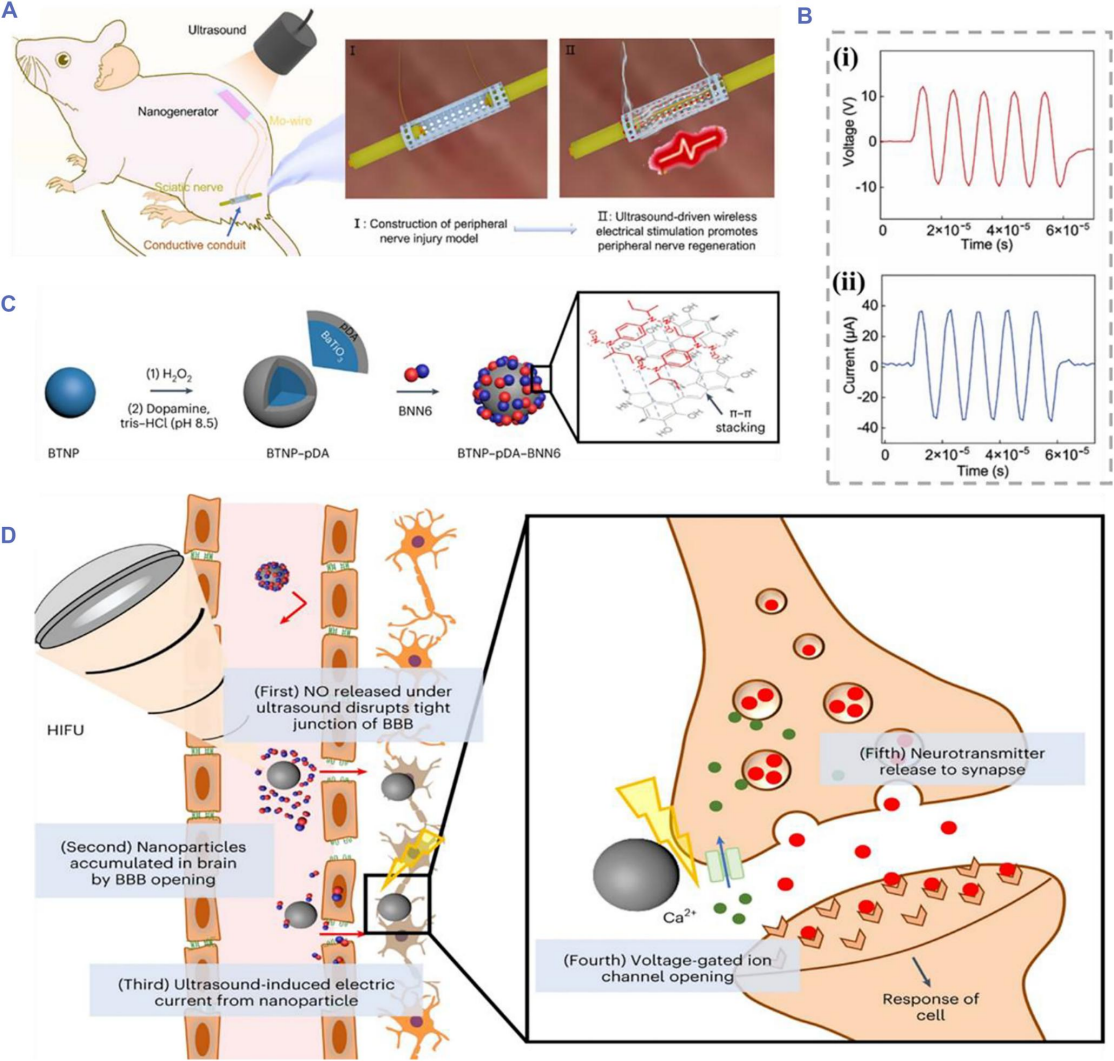
US‐driven piezoelectric effect. (A) US driving strategy of the biodegradable piezoelectric NGC. (B) The open circuit voltage (i) and short circuit current (ii) output under US excitation. *Source*: (A, B) Reproduced with permission.^51^ Copyright 2022, Elsevier. (C) Schematic diagram of the preparation of multifunctional piezoelectric nanoparticles. (D) US‐driven BTNP‐pDA‐BNN6 nanoparticles for local nerve stimulation. *Source*: (C, D) Reproduced with permission.^78^ Copyright 2023, The Authors, published by Springer Nature.

More recently, Li and Supponen et al. reported a hydrogel bioadhesive agent based on anchoring agent and chitosan hydrogel mediated by low‐frequency US (20–35 kHz) for tough bioadhesion to tissue interfaces and further indicated that US cavitation effect enhanced this adhesion property.^77^ In addition, the tissue penetration and mechanical vibration brought by US can be combined with piezoelectric materials to achieve wireless ES in vivo. As shown in Figure 3A,B, Luo and his coworkers prepared a biodegradable piezoelectric NGC, which could realize wireless ES in vivo under US excitation, and the generated voltage output was consistent with US frequency.^51^ In addition to the US‐driven piezoelectric effect, the US‐driven multifunction platforms combined the surface‐modified piezoelectric nanoparticles with US excitation have been constructed to increase the versatility of wireless piezoelectric devices. As an example, the multifunctional nanoparticles comprising N,N′‐di‐sec‐butyl‐N,N′‐dinitroso‐1,4‐phenylenediamine (BNN6) and BT nanoparticles with polydopamine (pDA) coating (called BTNP‐pDA‐BNN6 nanoparticles) were designed by Kim et al., which were used to relieve PD symptoms (Figure 3C).^78^ The mechanism of noninvasive penetration of blood‐brain‐barrier (BBB) and local ES under US excitation was further investigated (Figure 3D). These cutting‐edge applications illustrate that US has a bright future in wireless piezoelectric stimulation for neural tissue engineering.

### 4.2 Mechanical movement

**FIGURE 4:**
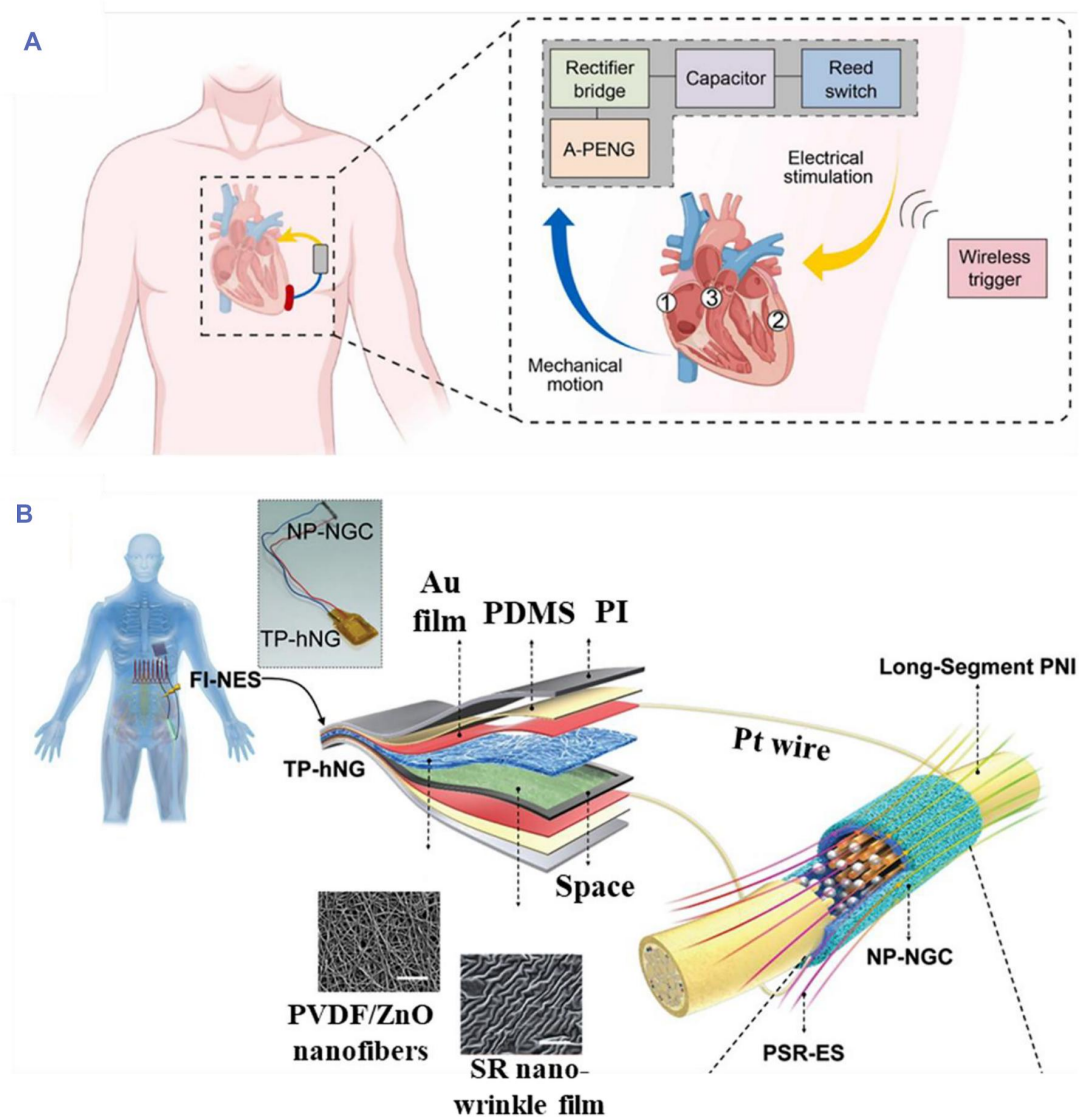
Mechanical movement‐driven piezoelectric effect. (A) Schematic diagram of the potassium sodium niobate ceramics nanomaterial‐based piezoelectric pacemaker opened by wireless trigger for cardiac pacing. *Source*: Reproduced with permission.^82^ Copyright 2022, Elsevier. (B) Working principle of a novel self‐powered system for nerve injury repair. *Source*: Reproduced with permission.^83^ Copyright 2021, John Wiley and Sons.

Benefiting from the direct piezoelectric effect of piezoelectric materials, a variety of mechanical energy in nature can be converted into electrical energy of piezoelectric materials, such as air flow, water flow, and motor vibration. Especially, miscellaneous mechanical movements of the human body, including muscle contraction and relaxation (skeletal muscle, heart, etc.), blood flow, middle ear tympanic membrane vibration, and small movements such as cell migration can cause deformation of piezoelectric materials in vivo to further achieve electrical signal output.^79^, ^80^, ^81^, ^82^, ^83^ By integrating the mechanical energy of human motion, piezoelectric materials can achieve wireless ES without other power equipment. For instance, Zhang et al. proposed a potassium sodium niobate ceramics nanomaterial‐based piezoelectric pacemaker, which could convert the bioenergy of the heart beating into electrical signal output to achieve effective myocardial pacing and conduction system pacing, as schemed in Figure 4A.^82^ In addition, in vivo piezoelectric devices can also collect the energy of mechanical movements of organs to supply power for the required tissues. Feng et al. designed a novel self‐powered system consisting of a triboelectric/piezoelectric nanogenerator and a multifunctional NGC, which could convert respiration‐generated biomechanical energy into electrical energy and transmit it to the sciatic nerve defect for promoting nerve regeneration (Figure 4B).^83^

## 5 NEUROENGINEERING APPLICATIONS OF PIEZOELECTRIC BIOMATERIALS

Piezoelectric biomaterials have exhibited unpredictable potential in various fields since their direct piezoelectric effects are able to overcome deficiencies of traditional ES equipment and perform wireless energy conversion under external mechanical stimulation. In particular, piezoelectric biomaterials possess outstanding features such as flexibility and biosafety, and the combination of multiple piezoelectric biomaterials enables them to efficiently adapt to human skin, soft tissue, and even nerve tissue. Therefore, piezoelectric biomaterials have received great attention in the field of local ES for nerve tissue engineering. In this section, we reviewed the latest progress of wireless ES based on piezoelectric biomaterials in central nerve repair and peripheral nerve repair.

### 5.1 Central nerve repair

**FIGURE 5:**
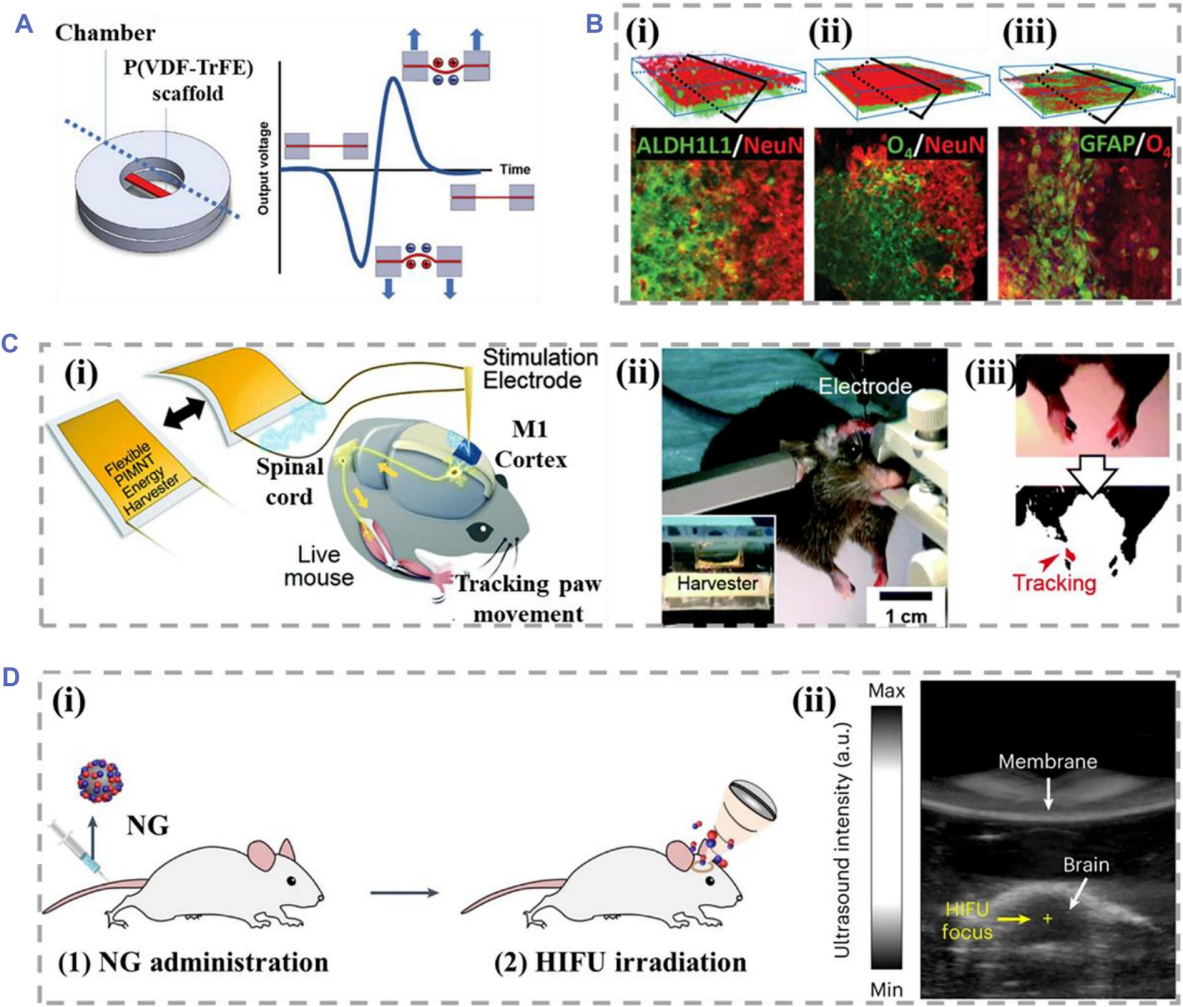
Piezoelectric biomaterials for CNS. (A) Schematic diagram of the electrospun P(VDF‐TrFE) nanofiber scaffold and generated piezoelectric effect driven by hydroacoustic actuation. (B) Confocal images of neural networks derived from human neural stem cells induced by P(VDF‐TrFE) nanofiber scaffold. NeuN is a neuronal marker, ALDH1L1 and O_4_ are markers for astrocytes and oligodendrocytes, and GFAP is an astrocyte marker. *Source*: (A, B) Reproduced with permission.^44^ Copyright 2021, John Wiley and Sons. (C) Schematic diagram (i) and photo (ii) of self‐powered DBS. (iii) Forelimb motion tracking. *Source*: Reproduced with permission.^80^ Copyright 2015, The Royal Society of Chemistry. (D) Aggregation of BTNP‐pDA‐BNN6 nanoparticles in brain tissue. Schematic diagram (i) and real‐time US‐guided images (ii) of nanoparticles administration. *Source*: Reproduced with permission.^78^ Copyright 2023, The Authors, published by Springer Nature. CNS, central nerve system; DBS, deep brain stimulation; P(VDF‐TrFE), poly(vinylidene fluoride‐trifluoro ethylene).

Several key factors after CNI occurrence, including inhibition factors in the tissue environment, poor regeneration ability of adult neurons, and aberrant synaptogenesis, make it difficult to regenerate injured nerves and recover central nerve system function.^84^ In order to develop a piezoelectric strategy for multifunctional neural tissue engineering, Nam et al. investigated the regulation of electrospun P(VDF‐TrFE) nanofiber scaffold driven by hydroacoustic actuation on neural stem cell differentiation (Figure 5A).^44^ The piezoelectric scaffold could output 200 mV_p‐p_ physiologically related potentials under the stimulation of 3 Hz sound waves, which could induce human neural stem cells to construct neural networks composed of neurons at the top layer and glial cells at the bottom layer, as shown in Figure 5B.^44^ In addition to study piezoelectricity of cell culture systems in vitro, Lee et al. realized the mediation of forearm movement in mice under deep brain ES without any external power supply. The flexible piezoelectric energy harvester derived from modified Pb‐based perovskite crystals could generate a maximum output current of 0.57 mA with mechanical deformation, enabling the control of living behavior under self‐powered deep brain stimulation (DBS) (Figure 5C).^80^

In addition to ES behavior control, similar DBS strategies caused by piezoelectric biomaterials are also used in the treatment of nervous system diseases. The C@BT piezoelectric nanoparticles reported by Shen et al. could generate electromagnetic fields under US driving, which has been proved to regulate neural plasticity and restore degenerated dopamine neurons.^29^ Furthermore, the PD zebrafish showed significant motor recovery and normal tyrosine hydroxylase expression level after DBS treatment with C@BT nanoparticles, suggesting the potential of piezoelectric nanoparticles in treating neurodegenerative diseases. However, such piezoelectric biomaterials require direct injection to the target treatment site, which brings difficulties to the surgical operation and patient recovery. To avoid this issue, Kim et al. systemically administrated the BTNP‐pDA‐BNN6 nanoparticles for the remission of PD symptoms. Under targeted high‐intensity focused US application, nitric oxide released from BTNP‐pDA‐BNN6 nanoparticles could cause a site‐specific transient BBB opening, which allowed the nanoparticles to accumulate into the nerve injury site. Besides, piezoelectrically induced electric currents could stimulate neurotransmitter release so as to treat neurological disorders, as shown in Figure 5D.^78^ Such results show the great potential of wireless self‐powered piezoelectric materials in nerve repair and brain‐computer interface.

### 5.2 Peripheral nerve repair

**FIGURE 6:**
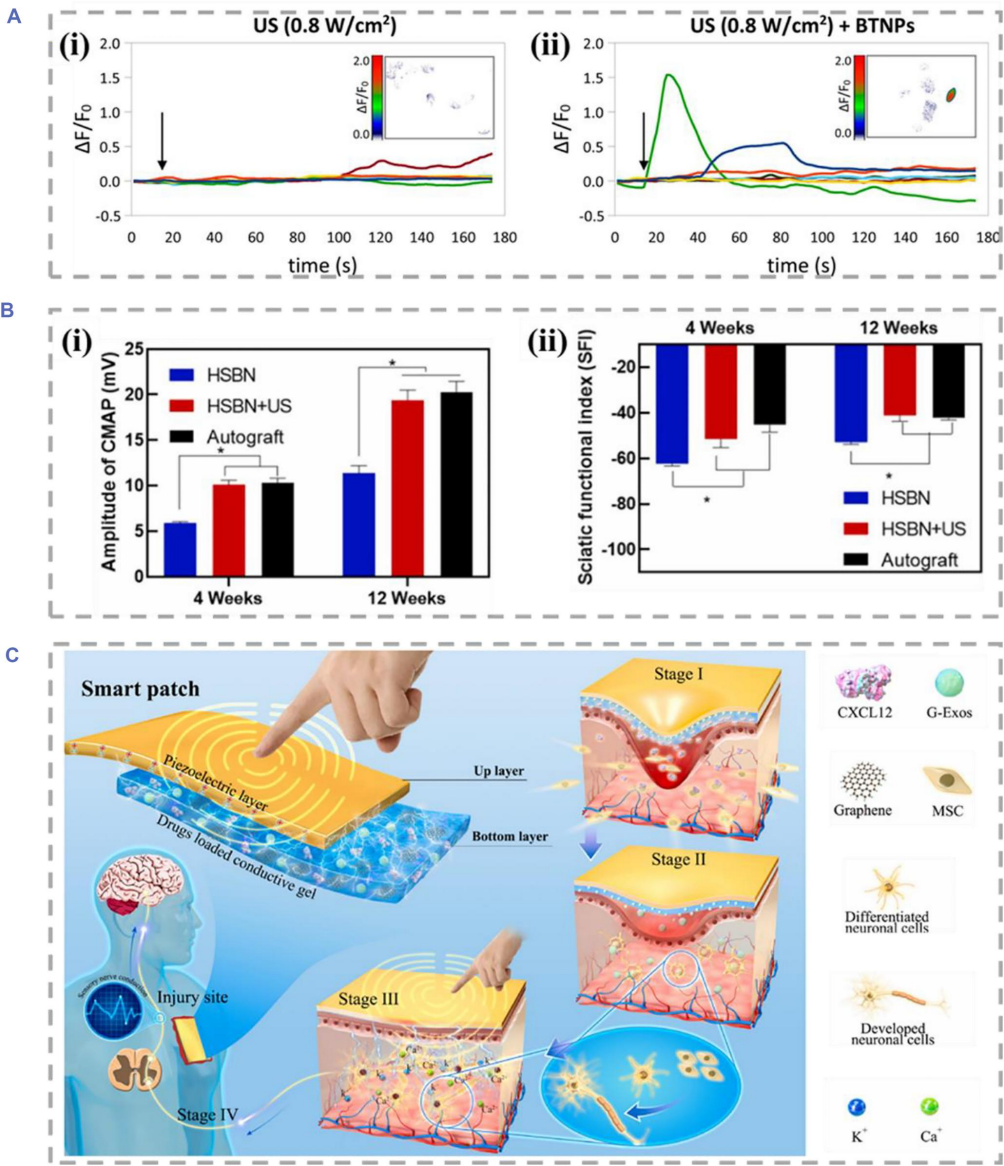
Piezoelectric biomaterials for PNS. (A) Calcium fluorescence intensity of SH‐SY5Y‐derived neurons in response to US (0.8 W/cm^2^) without (i) and with (ii) BT nanoparticles. *Source*: Reproduced with permission.^26^ Copyright 2015, American Chemical Society. (B) Peak amplitude of CMAPs (i) and SFI statistics (ii). *Source*: Reproduced with permission.^51^ Copyright 2022, Elsevier. (C) Schematic diagram of the PVDF‐based piezocomposite patch for skin nerve systems. *Source*: Reproduced with permission.^88^ Copyright 2022, Elsevier. CMAPs, compound muscle action potentials; PNS, peripheral nervous system; PVDF, poly(vinylidene fluoride); SFI, sciatic nerve function index.

Peripheral nervous system (PNS) is another important part of the nervous system, and PNI has received great attention in recent years. Compared with CNI, the neurons in PNS tissue environment have a stronger ability to regenerate and recover.^85^, ^86^ Recently, a variety of piezoelectric biomaterials have also been developed to promote PNI recovery. The work of Pané et al. has proved that the piezoelectric effect mediated by US of the PVDF membranes could promote the differentiation of PC12, which was comparable to that of neuronal growth factor.^87^ Furthermore, the local ES of piezoelectric biomaterials could cause changes in cell action potential. Ciofani et al. investigated the effect of BT nanoparticles on cell response under US. The results showed that the electrical signals from piezoelectric materials could cause changes in the intracellular calcium and sodium ion fluxes, which implied the stimulating effect of piezoelectric materials in nerve cells (Figure 6A).^26^

Up to date, piezocomposites have been further employed in the treatment of PNI in many cutting‐edge applications. The biodegradable piezoelectric nanogenerator reported by Luo et al. displayed a great therapeutic effect in the rat sciatic nerve defect models.^51^ As shown in Figure 6B, the amplitude of the compound muscle action potentials and sciatic nerve function indexes in the treatment group were similar to those in the autograft group, which showed that piezocomposites could be a strong candidate for autologous nerve transplantation in the treatment of PNI. In addition to providing wireless ES strategies instead of traditional NGCs for large gap nerve defect, the piezoelectric biomaterials could also be used to repair dense nerves as patches. For example, by integrating the piezoelectricity of modified PVDF and bioactive drug loading capacity of the conductive hydrogel, Peng et al. explored the therapeutic effect of PVDF‐based piezocomposite patches in the damaged skin nervous system (Figure 6C).^88^ In the treatment of the skin full‐excision models without skin nerve systems, the PVDF‐based patches with piezoelectricity and chemokines were confirmed to be effective in regenerating damaged neurons and restoring skin sensory function.

## 6 CONCLUSION AND OUTLOOK

To sum up, this review has summarized the recent progress on the piezoelectric biomaterials for neural tissue engineering. With the development of material science, engineering technology, and neural tissue, a variety of piezoelectric biomaterials have been imparted with numerous charming characteristics, such as electrical signal output matched with physiology, wireless ES, biocompatibility, and mechanical strength matching with soft tissues. Miscellaneous external mechanical fields have been introduced to stimulate their piezoelectricity, and such wireless self‐powered strategies driven by external fields possess bright application prospects in neural tissue engineering. These features have greatly promoted the frontier development of ES in neural tissue engineering and broadened the biomedical applications of piezoelectric biomaterials in neural diseases, DBS, and injured nerve repair. Nonetheless, there are still some issues about applying piezoelectric biomaterials in the field of neural tissue engineering to be explored in depth.

First, the novel piezoelectric biomaterials with adaptability and micro/nanostructures remained to be explored. In this regard, novel piezoelectric hydrogels could be expected as ideal implantation candidates to match the mechanical strength of soft tissues such as nerve tissue, thus providing local ES and effective transplantation for nerve tissue engineering. With the development of micro/nanomaterial science, biomaterials with micro/nanostructures are considered to provide surface morphology to promote the directional extension of neurites in the construction of functional neural networks and nerve injury repair. Surface morphology and micro/nanostructures of the composite piezoelectric materials can be realized based on the cutting‐edge engineering technologies such as surface modification, electrospinning, and 3D printing, which would respond to the specific needs in neural tissue engineering.

The second issue refers to the effective wireless ES application in nerve tissue engineering. Appropriate biodegradability is essential for NGCs used for nerve injury repair because NGCs should provide sufficient mechanical support as well as physical and chemical cues in the early stage of nerve injury repair while they are expected to degrade without surgical removal after nerve injury repair. From the perspective of local ES implantation equipment used for nerve tissue engineering, the piezoelectric biomaterials with appropriate biodegradability can generate wireless ES while avoiding unnecessary secondary surgery injury. Therefore, novel piezoelectric devices derived from biodegradable piezoelectric nanoparticles and organic polymer materials are still expected to be developed.

The third issue concerns the standardized mass production and personalized customization of piezoelectric biomaterials. As piezoelectric biomaterials have many applications in neural tissue engineering, personalized customization for special scenes is necessary. So far, the preparation of piezoelectric biomaterials for neural tissue engineering is still in the laboratory stage, lacking standardized and unified production processes, which limits the versatility and therapeutic effects of piezoelectric biomaterials in the application process. The standardization of piezoelectric materials used in neural tissue engineering requires the cooperation of researchers, medical device companies, and governments. Although challenges still exist, our review will give some inspiration to researchers to promote the progress of piezoelectric biomaterials in neural tissue engineering.

## AUTHOR CONTRIBUTIONS

Renjie Chai, Ling Lu and Fanglei Ye provided the idea; Dongyu Xu wrote the manuscript; Hui Zhang, Yu Wang and Yuan Zhang revised the manuscript.

## CONFLICT OF INTEREST STATEMENT

The authors declare no conflict of interest.
